# Supplementary material for: The effect of birth weight on body composition: Evidence from a birth cohort and a Mendelian randomization study
Source: PLoS One. 2019 Sep 10;14(9):e0222141. doi: 10.1371/journal.pone.0222141 (PMC6736493; doi:10.1371/journal.pone.0222141)
Supplement: S2 Table — (DOCX) [file pone.0222141.s002.docx]

S2 Table. Adjusted associations of birth weight, birth weight z-score and gestational age with body composition in complete case analysis in the Hong Kong’s “Children of 1997” birth cohort, Hong Kong, China, 1997 to 2016.

| Outcome | Exposure | Sex-adjusted as confounder | | p-value of interaction with sex | Boys | | Girls | |
| --- | --- | --- | --- | --- | --- | --- | --- | --- |
|  |  | Beta | 95% CI |  | Beta | 95% CI | Beta | 95% CI |
| Muscle mass (kg) | Birth weight (kg) | 2.43 | 2.00 to 2.86 | 0.08 | 2.80 | 2.06 to 3.54 | 2.03 | 1.61 to 2.44 |
|  | Birth weight z-score | 1.30 | 1.10 to 1.49 | 0.01 | 1.53 | 1.20 to 1.86 | 1.04 | 0.86 to 1.22 |
|  | Birth weight adjusted for gestational age | 3.33 | 2.83 to 3.84 | 0.02 | 3.91 | 3.04 to 4.78 | 2.70 | 2.21 to 3.19 |
|  | Gestational age (week) | 0.02 | -0.10 to 0.14 | 0.69 | 0.001 | -0.21 to 0.21 | 0.05 | -0.07 to 0.17 |
| Grip strength (kg) | Birth weight (kg) | 1.45 | 0.95 to 1.96 | 0.35 | 1.67 | 0.84 to 2.51 | 1.19 | 0.63 to 1.75 |
|  | Birth weight z-score | 0.70 | 0.48 to 0.93 | 0.43 | 0.78 | 0.40 to 1.15 | 0.60 | 0.35 to 0.85 |
|  | Birth weight adjusted for gestational age | 1.86 | 1.26 to 2.46 | 0.30 | 2.14 | 1.15 to 3.12 | 1.50 | 0.84 to 2.16 |
|  | Gestational age (week) | 0.06 | -0.08 to 0.20 | 0.97 | 0.07 | -0.16 to 0.30 | 0.06 | -0.09 to 0.22 |
| Fat percentage | Birth weight (kg) | 0.60 | 0.06 to 1.13 | 0.64 | 0.46 | -0.32 to 1.24 | 0.71 | -0.02 to 1.44 |
|  | Birth weight z-score | 0.40 | 0.16 to 0.64 | 0.95 | 0.40 | 0.05 to 0.75 | 0.39 | 0.06 to 0.72 |
|  | Birth weight adjusted for gestational age | 1.00 | 0.36 to 1.63 | 0.88 | 1.03 | 0.11 to 1.96 | 0.94 | 0.06 to 1.81 |
|  | Gestational age (week) | -0.06 | -0.21 to 0.08 | 0.27 | -0.15 | -0.36 to 0.07 | 0.02 | -0.18 to 0.22 |

Adjustment: second-hand and maternal smoking, highest parental education, parental occupation, household income, type of housing and sex.
