# Supplementary material for: The effect of birth weight on body composition: Evidence from a birth cohort and a Mendelian randomization study
Source: PLoS One. 2019 Sep 10;14(9):e0222141. doi: 10.1371/journal.pone.0222141 (PMC6736493; doi:10.1371/journal.pone.0222141)
Supplement: S3 Table — (DOCX) [file pone.0222141.s003.docx]

S3 Table. Single nucleotide polymorphisms (SNPs) independently predicted effects of maternal genetics net of infant genetics on birth weight in Europeans from the Early Growth Genetics (EGG) Consortium (p-value<5×10^-8^).

| SNP | Effect allele | Other allele | Effect allele frequency | Beta | Standard error | F-statistic for the jth variant^a^ | r2 |
| --- | --- | --- | --- | --- | --- | --- | --- |
| rs17037427 | A | C | 0.837 | -0.0339 | 0.0058 | 34 | 0.0003 |
| rs934232 | T | C | 0.4887 | -0.0249 | 0.0043 | 34 | 0.0003 |
| rs560887 | T | C | 0.2992 | -0.0401 | 0.0046 | 76 | 0.0007 |
| rs9855896 | A | G | 0.7817 | -0.0285 | 0.0051 | 31 | 0.0003 |
| rs6781704 | A | G | 0.5316 | 0.0377 | 0.0043 | 77 | 0.0007 |
| rs4679760* | C | G | 0.4154 | -0.0366 | 0.0044 | 69 | 0.0007 |
| rs6553731 | A | G | 0.5961 | -0.0245 | 0.0045 | 30 | 0.0003 |
| rs2946179 | T | C | 0.2627 | -0.0461 | 0.0049 | 89 | 0.0008 |
| rs34471628 | A | G | 0.9612 | 0.0643 | 0.0112 | 33 | 0.0003 |
| rs9379084 | A | G | 0.1168 | -0.0403 | 0.0068 | 35 | 0.0003 |
| rs6911024 | T | C | 0.9009 | 0.0416 | 0.0073 | 32 | 0.0003 |
| rs2971669 | T | C | 0.2169 | 0.0287 | 0.0052 | 30 | 0.0003 |
| rs148982377 | T | C | 0.9589 | -0.0722 | 0.0111 | 42 | 0.0004 |
| rs6995390* | A | T | 0.8364 | -0.0343 | 0.0058 | 35 | 0.0003 |
| rs10814916 | A | C | 0.4907 | -0.0244 | 0.0042 | 34 | 0.0003 |
| rs72760655 | A | C | 0.3205 | -0.0299 | 0.0047 | 40 | 0.0004 |
| rs10509669* | A | T | 0.7459 | 0.0357 | 0.0049 | 53 | 0.0005 |
| rs2168101 | A | C | 0.3096 | -0.0374 | 0.0048 | 61 | 0.0006 |
| rs111867185 | T | C | 0.1372 | 0.0358 | 0.0064 | 31 | 0.0003 |
| rs10830963* | C | G | 0.7224 | -0.046 | 0.0048 | 92 | 0.0008 |
| rs7122907 | T | G | 0.6589 | -0.0254 | 0.0046 | 30 | 0.0003 |
| rs6487930 | A | G | 0.515 | 0.0272 | 0.0043 | 40 | 0.0004 |
| rs180438 | A | G | 0.8068 | -0.0392 | 0.0054 | 53 | 0.0005 |
| rs17033114 | T | C | 0.9373 | 0.0605 | 0.0094 | 41 | 0.0004 |
| rs597808 | A | G | 0.4851 | -0.0336 | 0.0043 | 61 | 0.0006 |
| rs3784789* | C | G | 0.3317 | -0.029 | 0.0045 | 42 | 0.0004 |
| rs12909648 | A | G | 0.4769 | -0.0272 | 0.0042 | 42 | 0.0004 |
| rs7177338 | A | G | 0.5265 | 0.0265 | 0.0043 | 38 | 0.0004 |
| rs71367412 | T | C | 0.2135 | 0.0315 | 0.0053 | 35 | 0.0003 |
| rs2918301 | T | C | 0.1535 | -0.0445 | 0.006 | 55 | 0.0005 |

a the F-statistic for the jth variant is approximated as (${N-K-1}/K)* {(R^{2}}/{1-R^{2}}$)

* Palindromic SNPs, all were aligned with the outcome
