# Supplementary material for: The effect of birth weight on body composition: Evidence from a birth cohort and a Mendelian randomization study
Source: PLoS One. 2019 Sep 10;14(9):e0222141. doi: 10.1371/journal.pone.0222141 (PMC6736493; doi:10.1371/journal.pone.0222141)
Supplement: S4 Table — (DOCX) [file pone.0222141.s004.docx]

S4 Table. Single nucleotide polymorphisms (SNPs) with potential pleiotropic effects, and/or potential confounders from Ensembl, GWAS Catalog, PhenoScanner, and UK Biobank.

| SNPs | Gene nearby | Ensembl | GWAS Catalog | PhenoScanner (P<10E-5) | Potential pleiotropic and/or confounders in UK Biobank(P<10E-4) |
| --- | --- | --- | --- | --- | --- |
| rs17037427 | *CLCN6* | - | - | Blood pressure, Hypertention, Vascular or heart problems diagnosed, Medication for cholesterol, blood pressure or diabetes, Mean corpuscular hemoglobin, Mean corpuscular volume, Hip circumference, High cholesterol | - |
| rs934232 | *ZFP36L2* | - | - | Hematocrit, Hemoglobin concentration, Diastolic blood pressure | Height |
| rs560887 | *G6PC2* | Fasting glucose, Glycated hemoglobin, HOMA-B, Metabolic syndrome, Pluse pressure | Fasting glucose, Hemoglobin A1c, Metabolic levels, Metabolic syndrome, HOMA-B, Pluse preassure, Glycated hemoglobin levels | Fasting glucose and related traits, Metabolite levels, Hemoglobin A1c, Glycated hemoglobin levels, Glucose transporter type 2, HOMA-B, Metabolic syndrome, Glycemic traits, Sitting height, Systolic blood pressure | Height |
| rs9855896 | *LINC00690* | - | - | - | - |
| rs6781704 | *ADCY5* | - | - | Forced expiratory volume in 1-second, Body fat percentage, Leg fat percentage, Trunk fat percentage, Potassium in urine, Waist circumference, Forced vital capacity | - |
| rs4679760 | *KCNAB1* | - | - | - | - |
| rs6553731 | *RN7SKP13* | - | - | - | - |
| rs2946179 | *RP11-542A14.2* | - | - | Body fat (trunk, arm, leg) percentage, Whole body (leg, arm) fat mass, Impedance of whole body (arm, leg), Systolic blood pressure, Vascular or heart problems, Coronary artery disease | - |
| rs34471628 | *DUSP1* | - | C-reactive protein levels | Height, Crohns disease, No blood clot, Bronchitis, Emphysema, Asthma, Rhinitis, Eczema or allergy | Height, Income |
| rs9379084 | *RREB1* | Breast cancer, Heel bone mineral density, Type 2 diabetes | Breast cancer, Heel bone mineral density, Type 2 diabetes, Height | Height, Sitting height, Heel bone mineral density, Forced vital capacity, Diabetes, Whole body (trunk, arm, leg) fat-free mass, Comparative height size at age 10, Whole body water mass, Basal metabolic rate, Hemoglobin concentration, Forced expiratory volume in 1-second, Breast cancer, No blood clot, Bronchitis, Emphysema, Asthma, Rhinitis, Eczema or allergy | Height |
| rs6911024 | *MICA* | - | - | Self-reported hypothyroidism or myxoedema, Height, Treatment with levothyroxine sodium, Self-reported malabsorption or coeliac disease, No blood clot, Bronchitis, Emphysema, Asthma, Rhinitis, Eczema or allergy, Lymphocyte count, Sitting height, White blood cell count, Rheumatoid arthritis, Treatment with thyroxine product, Hayfever, Allergic rhinitis or eczema, Intestinal malabsorption, Comparative height size at age 10, Sum basophil neutrophil counts, Myeloid white cell count, Granulocyte count, Neutrophil count, Sum neutrophil eosinophil counts, Self-reported hyperthyroidism or thyrotoxicosis, Forced expiratory volume in 1-second, Cause of death: calculus of gallbladder without cholecystitis, Total cholesterol | Height |
| rs2971669 | *GCK* | Fasting glucose, Glycated hemoglobin, HOMA-B levels, Pluse pressure | Pulse pressure | Fasting glucose, HbA1c, HOMA B, Type 2 diabetes, Other symptoms and signs involving the urinary system, Forced vital capacity | - |
| rs148982377 | *ZNF789* | Sex hormone levels | Sex hormone levels | Dihydroepiandrosterone sulphate, Height, Sitting height, Sex hormone levels, Body mass index, Whole body (arm, leg) fat percentage, Heel bone mineral density, Waist circumference, Lumber spine bone mineral density, Forced vital capacity, Home area population density: Scotland large urban area, Forced expiratory volume in 1-second, Whole body fat mass | Height |
| rs6995390 | *ZFHX4* | - | - | Hair or balding pattern, Systolic blood pressure, Age at menarche | Age of menarche |
| rs10814916 | *GLIS3* | Type 2 diabetes | Type 2 diabetes | Fasting glucose, Type 2 diabetes, Knee pain, Hand grip strength | Height |
| rs72760655 | *COL27A1* | - | - | - | - |
| rs10509669 | *PLCE1* | - | - | Impedance of whole body (leg, arm) | - |
| rs2168101 | *LMO1* | Lim domain only-1 polymorphism, Sporadic neuroblastoma | Height, Sporadic neuroblastoma | Sporadic neuroblastoma, Sitting height, Whole body (trunk, arm, leg) fat-free mass, Whole body water mass, Basal metabolic rate, Weight, Height | Height |
| rs111867185 | *AGBL2* | - | Medication use (antiglaucoma preparations and miotics) | Vascular or heart problems, Hypertension, Lymphocyte count, Blood pressure, Height, Medication for cholesterol, blood pressure or diabetes: blood pressure medication, Eye problems or disorders: glaucoma, Monocyte percentage of white cells, Lymphocyte percentage of white cells, Treatment with blood pressure medication, Mean corpuscular volume | Height, Age of menarche |
| rs10830963 | *MTNR1B* | Acute insulin response, Corrected insulin response, Fasting blood glucose, Glucose homeostasis traits, Glycated hemolobin levels, HbA1c, HOMA-B levels, Incremental insulin, Insulin disposition, Insulin levels, Metabolite levels, Obesity-related traits, Pulse pressure, Type 2 diabetes, | Type 2 diabetes, Fasting glucose, Hemoglobin A1c, Glycated hemoglobin, Insulin, Insulin dispostion index, Metabolite levels, Acute insulin response, HOMA-B, Glucose homeostasis traits, Pulse pressure | Fasting glucose, Fasting glucose related traits, Insulin, HOMA B, HbA1c, Type 2 diabetes, log CIR, log CIR adjusted for ISI, log DI, Metabolite levels, Metabolism, Glucose homeostasis traits | - |
| rs7122907 | *YAP1* | - | - | Pulse rate, Qualifications: college or university degree, Impedance of arm right | - |
| rs6487930 | *IPO8* | - | - | Forced vital capacity, Forced expiratory volume in 1-second, Forced expiratory volume in 1-second, Whole body (Leg, arm trunk) fat-free mass, Whole body water mass, Height, Basal metabolic rate | Height |
| rs180438 | *SLC38A4* | - | - | Height, Oleic acid 18:1n9 | Height |
| rs17033114 | *LINC00485* | - | - | Bilateral oophorectomy, | - |
| rs597808 | *ATXN2* | Blood pressure * alcohol consumption interaction, Hypothyroidism, Smoking, Waist-hip ratio | Systemic lupus erythematosus, Blood pressure * alcohol consumption interaction, Hypothyroidism, Smoking, Mean corpuscular hemoglobin, Waist-hip ratio, Colorectal cancer or advanced adenoma | Height, Plateletcrit, Eosinophil count, Platelet count, Sum eosinophil basophil counts, further would be checked in the Phenoscanner | Height, Past smoking |
| rs3784789 | *CSK* | - | - | Blood pressure, Vascular or heart problems, Hypertension, Medication for cholesterol, blood pressure or diabetes: blood pressure medication, Monocyte count, Platelet distribution width, Treatment with bendroflumethiazide, Treatment with ramipril, Monocyte percentage of white cells, Creatinine in urine, Red blood cell count, Granulocyte percentage of myeloid white cells, Coronary artery disease, Hearing difficulty or problems with background noise, Hematocrit, Age-related macular degeneration | - |
| rs12909648 | *AKAP13* | Systolic blood pressure | Systolic blood pressure | Eosinophil percentage of granulocytes, Neutrophil percentage of granulocytes, Eosinophil percentage of white cells, Monocyte percentage of white cells | - |
| rs7177338 | *FES* | - | - | Vascular or heart problems, Hypertension, Coronary artery disease, Blood pressure, Treatment with blood pressure medication, Cardiovascular diseases related traits, further would be checked in the Phenoscanner | - |
| rs71367412 | *RAI1* | - | - | Self-reported anal fissure | - |
| rs2918301 | *AC092566.1* | - | - | Atopic dermatitis, Self-reported endometriosis | - |
