# Supplementary material for: The effect of birth weight on body composition: Evidence from a birth cohort and a Mendelian randomization study
Source: PLoS One. 2019 Sep 10;14(9):e0222141. doi: 10.1371/journal.pone.0222141 (PMC6736493; doi:10.1371/journal.pone.0222141)
Supplement: S5 Table — (DOCX) [file pone.0222141.s005.docx]

S5 Table. Estimates of the effect of genetically predicted birth weight (maternal effects net of infant effects) (per z-score) on body composition with and without potentially pleiotropic single nucleotide polymorphisms (SNPs) and potentially confounded SNPs using Mendelian randomization with different methodological approaches.

| Outcome | Sex | SNPs^a^ | IVW | | | | WM | | MR-Egger | | | MR-PRESSO outlier corrected | | |
| --- | --- | --- | --- | --- | --- | --- | --- | --- | --- | --- | --- | --- | --- | --- |
|  |  |  | Beta | 95% CI | I^2^ (p-value) | Sex interaction p-value | Beta | 95% CI | Beta | 95% CI | Intercept p-value | Beta | 95% CI | Sex interaction p-value |
| Fat-free Mass (kg) | All | 30 | 0.77 | 0.22 to 1.33 | 89.3% (<0.001) | 0.40 | 1.03 | 0.68 to 1.38 | 0.53 | -1.83 to 2.89 | 0.84 | 0.87 | 0.53 to 1.21 | 0.76 |
|  |  | 25 | 0.70 | 0.07 to 1.32 | 89.2% (<0.001) | 0.58 | 0.69 | 0.29 to 1.09 | -0.19 | -2.99 to 2.62 | 0.53 | 0.76 | 0.36 to 1.16 | 0.78 |
|  |  | 15 | 0.19 | -0.37 to 0.75 | 79.0% (<0.001) | 0.35 | 0.11 | -0.35 to 0.56 | 0.87 | -1.73 to 3.46 | 0.60 | 0.35 | -0.08 to 0.78 | 0.21 |
|  | Male | 30 | 0.97 | 0.24 to 1.71 | 80.9% (<0.001) | - | 1.01 | 0.45 to 1.56 | 0.78 | -2.36 to 3.91 | 0.90 | 0.79 | 0.25 to 1.33 | - |
|  |  | 25 | 0.88 | 0.03 to 1.73 | 81.7% (<0.001) | - | 0.86 | 0.23 to 1.49 | -0.18 | -4.00 to 3.65 | 0.58 | 0.68 | 0.05 to 1.31 | - |
|  |  | 15 | 0.19 | -0.51 to 0.89 | 58.4% (0.002) | - | 0.55 | -0.15 to 1.24 | 0.91 | -2.37 to 4.19 | 0.66 | 0.19^b^ | -0.51 to 0.89 | - |
|  | Female | 30 | 0.60 | 0.14 to 1.05 | 81.9% (<0.001) | - | 0.77 | 0.42 to 1.12 | 0.32 | -1.62 to 2.26 | 0.77 | 0.69 | 0.38 to 1.00 | - |
|  |  | 25 | 0.54 | 0.03 to 1.05 | 81.5% (<0.001) | - | 0.72 | 0.32 to 1.11 | -0.18 | -2.47 to 2.10 | 0.53 | 0.68 | 0.32 to 1.04 | - |
|  |  | 15 | 0.19 | -0.31 to 0.68 | 70.2% (<0.001) | - | 0.24 | -0.24 to 0.72 | 0.84 | -1.48 to 3.16 | 0.57 | 0.28 | -0.18 to 0.75 | - |
| Fat mass (kg) | All | 30 | 0.58 | 0.01 to 1.15 | 78.0% (<0.001) | 0.12 | 1.07 | 0.60 to 1.53 | 0.69 | -1.74 to 3.12 | 0.93 | 0.75 | 0.29 to 1.21 | 0.61 |
|  |  | 25 | 0.59 | -0.01 to 1.20 | 75.2% (<0.001) | 0.19 | 0.95 | 0.43 to 1.47 | 1.33 | -1.41 to 4.06 | 0.59 | 0.55 | 0.08 to 1.02 | 0.67 |
|  |  | 15 | 0.79 | -0.06 to 1.63 | 80.2% (0.07) | 0.55 | 1.07 | 0.46 to 1.67 | 2.63 | -1.23 to 6.48 | 0.34 | 1.12 | 0.64 to 1.60 | 0.75 |
|  | Male | 30 | 0.18 | -0.53 to 0.89 | 75.3% (<0.001) | - | 0.66 | 0.08 to 1.24 | -0.29 | -3.29 to 2.72 | 0.75 | 0.71 | 0.21 to 1.21 | - |
|  |  | 25 | 0.28 | -0.48 to 1.03 | 72.2% (<0.001) | - | 0.65 | -0.01 to 1.30 | 0.73 | -2.68 to 4.14 | 0.79 | 0.74 | 0.18 to 1.29 | - |
|  |  | 15 | 0.57 | -0.36 to 1.50 | 72.0% (<0.001) | - | 0.90 | 0.12 to 1.67 | 0.60 | -3.80 to 4.99 | 0.99 | 1.06 | 0.44 to 1.69 | - |
|  | Female | 30 | 0.93 | 0.31 to 1.55 | 59.4% (<0.001) | - | 1.33 | 0.66 to 1.99 | 1.53 | -1.11 to 4.17 | 0.64 | 0.92 | 0.33 to 1.50 | - |
|  |  | 25 | 0.88 | 0.17 to 1.58 | 59.9% (<0.001) | - | 0.93 | 0.18 to 1.68 | 1.83 | -1.35 to 5.00 | 0.55 | 0.85 | 0.20 to 1.51 | - |
|  |  | 15 | 0.98 | -0.003 to 1.97 | 68.4% (<0.001) | - | 1.30 | 0.36 to 2.24 | 4.37 | 0.11 to 8.63 | 0.11 | 0.98 | 0.07 to 1.89 | - |
| Left grip strength (kg) | All | 30 | 0.58 | 0.12 to 1.04 | 77.5% (<0.001) | 0.22 | 0.56 | 0.20 to 0.93 | 1.13 | -0.82 to 3.07 | 0.57 | 0.65 | 0.30 to 1.01 | 0.07 |
|  |  | 25 | 0.55 | 0.01 to 1.09 | 79.3% (<0.001) | 0.31 | 0.66 | 0.25 to 1.08 | 0.60 | -1.85 to 3.06 | 0.96 | 0.65 | 0.24 to 1.05 | 0.13 |
|  |  | 15 | 0.41 | -0.22 to 1.03 | 76.1% (<0.001) | 0.66 | 0.54 | 0.07 to 1.00 | 0.54 | -2.41 to 3.49 | 0.93 | 0.52 | 0.12 to 0.92 | 0.56 |
|  | Male | 30 | 0.81 | 0.26 to 1.36 | 53.3% (<0.001) | - | 0.97 | 0.38 to 1.55 | 1.74 | -0.58 to 4.06 | 0.42 | 0.91 | 0.39 to 1.43 | - |
|  |  | 25 | 0.78 | 0.11 to 1.46 | 60.0% (<0.001) | - | 0.80 | 0.12 to 1.47 | 1.55 | -1.49 to 4.59 | 0.61 | 0.90 | 0.26 to 1.53 | - |
|  |  | 15 | 0.57 | -0.21 to 1.36 | 54.5% (0.006) | - | 0.61 | -0.16 to 1.38 | 0.77 | -2.93 to 4.47 | 0.91 | 0.55 | -0.03 to 1.13 | - |
|  | Female | 30 | 0.36 | -0.09 to 0.81 | 70.9% (<0.001) | - | 0.21 | -0.18 to 0.60 | 0.58 | -1.34 to 2.50 | 0.82 | 0.35 | 0.01 to 0.68 | - |
|  |  | 25 | 0.34 | -0.17 to 0.84 | 70.0% (<0.001) | - | 0.26 | -0.17 to 0.69 | -0.21 | -2.47 to 2.05 | 0.63 | 0.42 | 0.05 to 0.80 | - |
|  |  | 15 | 0.25 | -0.31 to 0.82 | 63.3% (<0.001) | - | 0.21 | -0.30 to 0.72 | 0.33 | -2.32 to 2.98 | 0.95 | 0.28 | -0.17 to 0.72 | - |
| Right grip strength (kg) | All | 30 | 0.58 | 0.10 to 1.06 | 79.4% (<0.001) | 0.43 | 0.72 | 0.33 to 1.11 | 1.27 | -0.76 to 3.30 | 0.49 | 0.69 | 0.29 to 1.09 | 0.52 |
|  |  | 25 | 0.54 | -0.02 to 1.10 | 80.6% (<0.001) | 0.58 | 0.79 | 0.34 to 1.25 | 0.56 | -1.98 to 3.09 | 0.99 | 0.60 | 0.15 to 1.04 | 0.93 |
|  |  | 15 | 0.35 | -0.25 to 0.95 | 74.2% (<0.001) | 0.95 | 0.60 | 0.09 to 1.10 | 1.12 | -1.69 to 3.93 | 0.58 | 0.49 | -0.001 to 0.98 | 0.78 |
|  | Male | 30 | 0.74 | 0.13 to 1.34 | 61.5% (<0.001) | - | 0.91 | 0.30 to 1.52 | 2.36 | -0.14 to 4.86 | 0.19 | 0.73 | 0.18 to 1.29 | - |
|  |  | 25 | 0.67 | -0.04 to 1.37 | 63.5% (<0.001) | - | 0.86 | 0.16 to 1.55 | 1.57 | -1.59 to 4.74 | 0.57 | 0.54 | -0.13 to 1.21 | - |
|  |  | 15 | 0.46 | -0.30 to 1.22 | 51.7% (0.01) | - | 0.85 | 0.08 to 1.62 | 2.68 | -0.68 to 6.04 | 0.19 | 0.62 | -0.06 to 1.30 | - |
|  | Female | 30 | 0.43 | -0.02 to 0.88 | 70.1% (<0.001) | - | 0.40 | -0.01 to 0.81 | 0.31 | -1.59 to 2.22 | 0.90 | 0.51 | 0.15 to 0.87 | - |
|  |  | 25 | 0.43 | -0.10 to 0.96 | 73.0% (<0.001) | - | 0.46 | -0.01 to 0.93 | -0.31 | -2.70 to 2.08 | 0.53 | 0.54 | 0.11 to 0.96 | - |
|  |  | 15 | 0.25 | -0.37 to 0.87 | 69.2% (<0.001) | - | 0.40 | -0.17 to 0.97 | -0.22 | -3.13 to 2.69 | 0.75 | 0.41 | -0.13 to 0.94 | - |

IVW: inverse variance weighting; WM: weighted median; MR-PRESSO: Mendelian Randomization Pleiotropy RESidual Sum and Outlier

a SNP= 30: all SNPs; SNP= 25, excluding maternal genotype related SNPs, and potential pleiotropic SNPs from GWAS catalog and Ensembl: rs560887 (*G6PC2*), rs2971669 (*GCK*), rs148982377 (*ZNF789*), rs2168101 (*LMO1*), rs10830963 (*MTNR1B*); excluding potential pleiotropic and/or confounded SNPs in UK Biobank in Bonferroni corrected significance (p-value<1×10^-4^) and in PhenoScanner (p-value<1×10^-5^): rs934232 (*ZFP36L2*), rs34471628 (*DUSP1*), rs9379084 (*RREB1*), rs6911024 (*MICA*), rs6995390 (*ZFHX4*), rs10814916 (*GLIS3*), rs111867185 (*AGBL2*), rs6487930 (*IPO8*), rs180438 (*SLC38A4*), rs597808 (*ATXN2*).

b No outlier is found, presenting the raw estimate instead.
