# Supplementary material for: The effect of birth weight on body composition: Evidence from a birth cohort and a Mendelian randomization study
Source: PLoS One. 2019 Sep 10;14(9):e0222141. doi: 10.1371/journal.pone.0222141 (PMC6736493; doi:10.1371/journal.pone.0222141)
Supplement: Attachment — Submitted filename: Response to Reviewers_Finalized.docx [file pone.0222141.s006.docx]

**Manuscript PLOS ONE**

**PONE-D-19-16847**

**Title: The effect of birth weight on body composition: Evidence from a birth cohort and a Mendelian randomization study**

**Responses to Editors’ and Reviewers’ comments**

# Reviewer 1 #

It was a pleasure to review this comprehensive study performed by Liu and colleagues, investigating the effect of birth weight on consequent body composition. The authors are experienced with the applied methods and have taken care in the performing appropriate analyses.

## **C1.1**

The work is already close to a standard suitable for publication. My main suggestion for improvement would be some clarification regarding the interpretation of causality. I think it is important that the authors discuss the potential implications of a shared (genetic) aetiology between birth weight and consequent body composition. Keeping this in mind, is it justified that the authors should imply in their conclusion that birth weight has a causal effect on consequent body composition?

*Thank you very much indeed for this comment. We have been inspired to rethink exactly how we are proxying birth weight, so as to emphasis that we are not looking at shared genetic etiology of birth weight and body composition, but are using a genetic predictor of maternal effects on birth weight. As such, we now used genetic instruments for maternal only effects on birth weight. Birth weight was considered as a proxy of maternal intrauterine environment. The new analysis gives similar results and is consistent with our observational study from a setting with little socio-economic patterning of obesity, i.e., from a setting less open to confounding by socio-economic position than more commonly studies Western populations.*

## **C1.2**

In any case, I think that the authors should also be more clear about whether they are genuinely suggesting birth weight as a target for clinical intervention - although they do state this in the last line of the conclusion, I must admit that I'm struggling to take it seriously. The practicalities of modifying birth weight is another consideration. How might this be achieved? If the authors prefer not to answer this, then they should probably remove this suggestion.

*Thank you for your comment. Please accept our apologies for being unclear. We agree that birth weight might not be a practical intervention. In fact, birth weight is difficult to change[1] and does not have an “optimal” definition.[2] Instead, etiologically, this study focused on testing and elucidating a potential role of muscle mass, as a target, on the pathway from lower birth weight to higher diabetes risks,[3] given the association of lower fat-free mass with higher diabetes risk[4] has been confirmed in an MR study. From a public health perspective of potential interventions, we suggested increasing fat-free mass (muscle mass) as a potential and modifiable target in the reducing risk of diabetes, providing birth weight is less likely be modified.*

*We have added these points as below.*

***(Introduction-1^st^ paragraph)***

**From:**

**Elucidating the pathways linking birth weight with diabetes may provide additional insights into the identification of intervention targets.**

**To:**

**Elucidating the pathways linking birth weight with diabetes may provide additional insights into the identification of intervention targets, since birth weight is difficult to change[1] and does not have an “optimal” definition.[2]**

***(Discussion-3^rd^ paragraph)***

**From:**

**From an etiological perspective, a causal association of birth weight with muscle mass provides a potential mechanistic, a modifiable pathway from lower birth weight to higher diabetes risks.[3, 5, 6]**

**To:**

**From an etiological perspective, a causal association of birth weight with muscle mass provides a potential mechanistic, a modifiable pathway from lower birth weight to higher diabetes risks.[3, 5, 6]. Given birth weight is difficult to change, such findings suggest that muscle building might reduce diabetes risk due to lower birth weight.**

## **C1.3**

Some discussion about the range of birth weights for which these findings might apply is also warranted. Can these estimates be extrapolated to the extremes of birth weight, for example?

*Thank you for your comment, which raises several important issues.*

*We agree that we didn’t examine the effect of birth weight on body composition among the participants with extreme values of birth weight, for the following reasons:*

1) *We would normally expect causal factors to act consistently unless there is a good reason to expect that the relevance of the specific operating mechanism varies by birth weight.[7]*
2) *Observationally, stratifying by birth weight (<2500g) might not have enough power to obtain the associations of interest, since only 155 of the participants included have birth weight lower than 2500g in the “Children of 1997” birth cohort.*

*Two-sample MR relies on summary data so, it is not possible to assess differences by subgroup of birth weight.*

*We have added these points as below.*

***(Discussion-3^rd^ paragraph)***

**From:**

**Asians have more than double the risk of developing diabetes than Europeans at the same BMI.[8]**

**To:**

**Asians have more than double the risk of developing diabetes than Europeans at the same BMI.[8] However, it is possible that the observed associations do not extend to the extremes of the birth weight distribution, where birth weight may be a symptom of specific pathology. Given this is likely to be rare, we do not have sufficient sample size to assess this possibility.**

## **C1.4**

Finally, I wonder whether the authors might also consider replacing (or supplementing) some of the existing results tables with figures (e.g. forest plots of the estimates and their confidence intervals). The current format used for presenting the data is a little overwhelming in places and therefore difficult to follow. Figures might make things easier to digest.

*Thank you for your comment.*

*We have extracted the MR-PRESSO estimates into the forest plots to present the estimates given it detects and corrects for pleiotropic outliers.*

***(Results-Mendelian randomization)***

**Figure 3. Mendelian randomization estimates of the effect of genetically predicted birth weight (maternal effects net of infant effects) (per z-score) on body composition with and without potentially pleiotropic single nucleotide polymorphisms (SNPs) and potentially confounded SNPs using MR-PRESSO.**

SNP= 30: all SNPs; SNP= 25, excluding maternal genotype related SNPs, and potential pleiotropic SNPs from GWAS catalog and Ensembl: rs560887 (*G6PC2*), rs2971669 (*GCK*), rs148982377 (*ZNF789*), rs2168101 (*LMO1*), rs10830963 (*MTNR1B*); excluding potential pleiotropic and/or confounded SNPs in UK Biobank in Bonferroni corrected significance (p-value<1×10^-4^) and in PhenoScanner (p-value<1×10^-5^): rs934232 (*ZFP36L2*), rs34471628 (*DUSP1*), rs9379084 (*RREB1*), rs6911024 (*MICA*), rs6995390 (*ZFHX4*), rs10814916 (*GLIS3*), rs111867185 (*AGBL2*), rs6487930 (*IPO8*), rs180438 (*SLC38A4*), rs597808 (*ATXN2*).

MR-PRESSO: Mendelian randomization pleiotropy residual sum and outlier

# Reviewer 2 #

The authors examined the associations of birth weight (BW) with muscle and fat mass in an observational study. They also performed a Mendelian Randomisation (MR) of BW on these measures in the UK Biobank study. I have several major concerns relating to the MR section of the paper.

## **C2.1**

The authors do not clearly state the question that they are seeking to answer with the BW-fat/muscle MR analysis. It would be helpful if the authors could provide a diagram (eg. a DAG) explaining the causal question which they seek to answer. For example, are the authors using birth weight as a proxy for intrauterine exposures which might influence muscle and fat mass? If so, the maternal birth weight-associated alleles that are not passed to the child are the appropriate instrumental variables to use. The issue of MR in this context was discussed in the latest BW GWAS paper (https://doi.org/10.1038/s41588-019-0403-1). Further papers discussing this issue are https://doi.org/10.12688/wellcomeopenres.10567.1 and https://doi.org/10.1093/ije/dyz019. Alternatively, the authors might be asking about whether the genetic variation underlying birth weight is shared with the underlying muscle and fat mass, in which case, the fetal genotype is more important. Could the authors please clarify this.

*Thank you for asking, given we are interested in interventions, we are trying to determine if birth weight is affected by endogenous maternal factors. As such, we have updated the MR analyses to assess the effect of maternal genetics on birth weight allowing for infant genetics using single nucleotide polymorphisms (SNPs) in Europeans from the Early Growth Genetics (EGG) Consortium.[9] We have added the corresponding DAG as Figure 1 in the* ***Introduction-3^rd^ paragraph.***

**Figure 1.** **Directed acyclic graph of the observational analysis and the Mendelian randomization analysis**

## **C2.2**

A major problem in MR with birth weight using fetal genetic instruments as the exposure is the violation of MR assumptions by the association of maternal genotype with the outcome of interest. The latest BW GWAS paper discovered additional BW associated SNPs and also quantified the independent maternal and fetal associations with BW at each of the loci. Ideally this larger list and independent fetal effect sizes should be used for the exposure SNPs, the summary statistics from these analyses adjusted for maternal/fetal genotype associations are available to download from the EGG consortium website http://egg-consortium.org/birth-weight-2019.html. These adjusted association statistics correct the SNP-exposure associations for maternal genetic effects. This still would not get around the problem of the potential violation of the MR assumptions, indeed the large attenuation of the association results when potentially maternal SNPs are excluded from the analyses suggests that failing to account for maternal effects may be affecting the results. To properly perform these analyses the SNP-outcome associations should be corrected for maternal genetic associations. If this is not possible, this limitation should be clearly stated and fully discussed in the paper. The confounding introduced by maternal genotype is briefly mentioned at the end of the limitations section, but the potential impact of this on the results and the interpretation of them is not given sufficient attention and need to be carefully discussed and made more prominent.

*Thank you very much indeed for this very helpful comment. We have carefully rethought.*

*Given a most recent GWAS from the EGG consortium has decomposed the contributions of direct fetal and indirect maternal genetic effects on birth weight,[9] we have updated the MR analyses to use genetic variants predicting maternal effects on offspring birth weight independent of fetal effects on birth weight to obtain the association of birth weight with body composition. These updated estimates are consistent with the previously positive estimates of birth weight with body composition.*

*We have added these points as below.*

*(Abstract)*

**From:**

**Using MR, birth weight was positively associated with fat-free mass (0.77 kg per birth weight z-score, 95% CI 0.17 to 1.36) but less clearly with fat mass (0.91, 95% CI -0.15 to 1.97). No difference by sex was evident.**

**To:**

**Using MR, birth weight was positively associated with fat-free mass (0.77 kg per birth weight z-score, 95% CI 0.22 ~~0.17~~ to 1.33 ~~1.36~~) ~~but less clearly with~~ and fat mass (0.58 ~~0.91~~, 95% CI 0.01~~-0.15~~ to 1.15 ~~1.97~~). No difference by sex was evident.**

*(Introduction-3^rd^ paragraph)*

**From:**

**Second, using an MR design, we validated our findings, assessing the effects of genetically predicted birth weight on body composition (fat-free mass, grip strength, and fat mass) in the largest publicly available genome wide association study (GWAS)**

**To:**

**Second, using an MR design, we validated our findings, by assessing the ~~effects~~ associations of ~~genetically predicted~~ birth weight predicted by maternal genetics independent of fetal genetics, as a proxy of the maternal intrauterine environment, on body composition (fat-free mass, grip strength, and fat mass) in the largest publicly available genome wide association study (GWAS)**

***(****Material and methods-Exposure- genetic predictors of* ***maternal only effect on*** *birth weight****)***

**From:**

**Single nucleotide polymorphisms (SNPs) predicting birth weight (sex-specific z-score) at genome-wide significance (p-value<5×10^-8^) adjusted for study-specific covariates and gestational age, where available, were obtained from a GWAS with genomic control comprising 153,781 individuals from 37 studies excluding non-singletons and premature births, who were 93% European, with mean birth weight 3415 grams and 46.9% women.[10]**

**To:
Single nucleotide polymorphisms (SNPs) predicting maternal effects on birth weight independent of fetal effects (~~sex-specific~~ z-score transformed) at genome-wide significance (p-value<5×10^-8^) adjusted for gestational age where available (only available in <15% of the sample) and study-specific covariates ~~and gestational age, where available,~~ were obtained from a GWAS consisting of two components, the Early Growth Genetics (EGG) Consortium (n=12,319, 10 studies in the EGG consortium of European descent imputed up to the HapMap 2 reference panel, and n=7,542, 2 studies in of European descent imputed up to the HRC panel) and the UK Biobank (n= 190,406, white European). A structural equation model was used to decompose the contributions of maternal and fetal genetic effects on birth weight (264,498 individuals own birth weight and 179,360 individuals offspring birth weight).[11] ~~with genomic control comprising 153,781 individuals from 37 studies excluding non-singletons and premature births, who were 93% European, with mean birth weight 3415 grams and 46.9% women.[10]~~**

**From:**

**We also checked for potential pleiotropic effects and confounding of these SNPs** **from the Bonferroni corrected significance (13 traits × 46 SNPs,** **p-value<1×10^-4^) of their associations with alcohol consumption (past and current), smoking (past and current), physical activity (light, moderate, and vigorous), socioeconomic position (income and education), age of voice braking, age of menarche, height, and birth weight of first child in the UK Biobank summary statistics.[12] SNPs associated with maternal genetic effects on offspring birth weight were also identified from the exposure GWAS[10] and a GWAS of offspring birth weight with maternal genetic effects.[13]**

**To:**

**We also checked for potential pleiotropic effects and confounding of these SNPs from the Bonferroni corrected significance (~~13~~ 12 traits × ~~46~~ 30 SNPs, p-value<1×10^-4^) of their associations with alcohol consumption (past and current), smoking (past and current), physical activity (light, moderate, and vigorous), socioeconomic position (income and education), age of voice braking, age of menarche, and height~~, and birth weight of first child~~ in the UK Biobank summary statistics.[12] ~~SNPs associated with maternal genetic effects on offspring birth weight were also identified from the exposure GWAS[10] and a GWAS of offspring birth weight with maternal genetic effects.[13]~~**

*(Material and methods-Sensitivity analyses relevant to Mendelian randomization)*

**From:**

**As sensitivity analyses, we excluded SNPs which may be invalid. These included 1) un-aligned palindromic SNPs; 2) SNPs associated with maternal genotype from GWAS studies[10, 13] and SNPs with potentially pleiotropic effects on muscle or fat given in Ensembl or the GWAS Catalog; 3) SNPs associated with height in the UK Biobank at genome-wide significance; 4) SNPs associated with potential confounders and/or pleiotropic effects in the UK Biobank at Bonferroni corrected significance and in PhenoScanner.**

**To:**

**As sensitivity analyses, we excluded SNPs which may be invalid. These included 1) ~~un-aligned palindromic SNPs; 2)~~ SNPs associated with ~~maternal genotype from GWAS studies[10, 13] and SNPs with~~ potentially pleiotropic effects on muscle or fat given in Ensembl or the GWAS Catalog; ~~3) SNPs associated with height in the UK Biobank at genome-wide significance; 4)~~ 2) SNPs associated with potential confounders and/or pleiotropic effects in the UK Biobank (p-value<1×10^-4^) at Bonferroni corrected significance and in PhenoScanner** **(p-value <1×10^-5^).**

*(Results-Mendelian randomization-Genetic instruments for* ***maternal only effects on*** *birth weight)*

**From:**

**Altogether, 60 SNPs predicted birth weight (p-value<5×10^-8^), of which 46 SNPs independently predicted birth weight in people of European ancestry.[10] The average of SNP-specific *F* statistics was 55, and all were >20 (S3 Table); the variance explained (r^2^) was 0.017. As such the MR study had 80% power with 5% alpha to detect a difference of 0.4 of an effect size in fat-free mass and fat mass per z-score of birth weight.**

**Of the 46 SNPs predicting birth weight, 2 were excluded because they were palindromic and could not be aligned, (rs6040076 (*JAG1*) and rs2150052 (*LPAR1*)) (S4 Table). Of the remaining 44 SNPs, 7 were related to maternal genetic effects on birth weight or had potentially pleiotropic effects, i.e., (height, and insulin response) in Ensembl or the GWAS Catalog. Of the remaining 37 SNPs, 12 SNPs were genome-wide significant for height in the UK Biobank. Of these 25 SNPs, 11 remained after excluding SNPs related to height, menarche, birth weight of the first child, and basal metabolic rate in the UK Biobank (p-value<1×10^-4^) and in PhenoScanner.**

**To:**

**Altogether, ~~60~~ 30 SNPs independently predicted effects of maternal genetics net of infant genetics on birth weight (p-value<5×10^-8^), ~~of which 46 SNPs independently predicted birth weight~~ in people of European ancestry.[10] The average of SNP-specific *F* statistics was ~~55~~ 79, and all were >~~20~~ 30 (S3 Table); the variance explained (r^2^) was ~~0.017~~ 0.013. As such, the MR study had 80% power with 5% alpha to detect a difference of ~~0.4~~ 0.04 of an effect size in fat-free mass and fat mass per z-score of birth weight.**

**Of the ~~46~~ 30 SNPs predicting birth weight, ~~2 were excluded because they all the~~ ~~were~~ 5 palindromic SNPs ~~and could not be~~ were aligned (S3 Table), ~~(rs6040076 (~~*~~JAG1~~*~~) and rs2150052 (~~*~~LPAR1~~*~~)) (S4 Table). Of the remaining 44 SNPs, 7 were related to maternal genetic effects on birth weight or~~ 5 SNPs had potentially pleiotropic effects, i.e., (height, and metabolic** **response ~~insulin response~~) in Ensembl or the GWAS Catalog. Of the remaining 25 SNPs ~~37 SNPs, 12 SNPs were genome-wide significant for height in the UK Biobank. Of these 25 SNPs, 11~~ 15 remained after excluding SNPs related to height, menarche, ~~birth weight of the first child,~~ income, and basal metabolic rate in the UK Biobank (p-value<1×10^-4^) and in PhenoScanner (p-value <1×10^-5^) (S4-S5 Tables).**

***(Results-Mendelian randomization estimates)***

**From:**

**Based on all 46 SNPs, genetically predicted birth weight was positively associated with fat-free mass and grip strength with stronger associations in men. After excluding all 35 potentially pleiotropic or confounded SNPs, the positive associations remained, although sex interaction was becoming unclear. Weighted median, MR-Egger, and MR-PRESSO gave similar estimates, although MR-Egger had wider confidence intervals (Table 3 and Table 4).**

**Table 5 shows that genetically predicted birth weight was associated with higher fat mass. No sex differences were evident. However, estimates were attenuated when all 35 potentially pleiotropic or confounded SNPs were excluded.**

**To:**

**Based on all ~~46~~ 30 SNPs, genetically predicted birth weight (maternal effects net of infant effects) was positively associated with fat-free mass, fat mass, and grip strength. No sex differences were evident ~~with stronger associations in men~~. After excluding 5 ~~all 35~~ potentially pleiotropic ~~or confounded~~ SNPs, the positive associations remained, however, the associations were not robust after additionally excluding 10 potentially pleiotropic and confounded SNPs (S5 Table).~~although sex interaction was becoming unclear. Weighted median, MR-Egger, and~~ Detecting and correcting for pleiotropic outliers, MR-PRESSO indicated robust positive estimates in particular with fat mass (Fig 3).~~gave similar estimates, although~~ MR-Egger had wider confidence intervals but had no indication of potential pleiotropy (~~S3 Table 3 and~~ S5 Table).**

**~~Table 5 shows that genetically predicted birth weight was associated with higher fat mass. No sex differences were evident. However, estimates were attenuated when all 35 potentially pleiotropic or confounded SNPs were excluded.~~**

***(Discussion-1^st^ paragraph)***

**From:**

**Using two different designs, with different assumptions and data sources, we found consistent evidence that birth weight was positively associated with muscle mass (fat-free mass) and grip strength but the positive association of birth weight with fat percentage (fat mass) was not so robust. These findings are consistent with previous observational studies,[14-16] but add by validating these observations in a setting with little socioeconomic patterning of birth weight and the use of MR.** **Our study also suggests birth weight may have a stronger effect on muscle mass (fat-free mass) in men than in women.**

**To:**

**Using two different designs, with different assumptions and data sources, we found consistent evidence that birth weight was positively associated with muscle mass (fat-free mass) ~~and~~ grip strength and ~~but the positive association of birth weight with~~ fat percentage (fat mass) ~~was not so robust~~. These findings are consistent with previous observational studies,[14-16] but add by validating these observations in a setting with little socioeconomic patterning of birth weight and the use of MR. ~~Our study also suggests birth weight may have a stronger effect on muscle mass (fat-free mass) in men than in women.~~**

***(Discussion-2^nd^ paragraph)***

**From:**

**MR-PRESSO also gave positive sex-specific estimates after taking potential pleiotropy into account.**

**To:**

**MR-PRESSO also gave consistently positive sex-specific estimates after taking potential pleiotropy into account (Fig 3).**

**From:**

**Fourth, the overlap of the GWAS of birth weight with UK Biobank is ~20%, nevertheless, the *F* statistic was 55 suggesting weak instrument bias is less likely.[17]**

**To:**

**Fourth, the overlap of the GWAS of birth weight with UK Biobank is ~~~20%~~ 90%, which might bias estimates towards the exposure-outcome association, nevertheless, the *F* statistic was ~~55~~ 79 suggesting weak instrument bias is less likely.[17]**

**From:**

**Eighth, MR provides an estimate of the effect of lifetime exposure rather than indicating the exact size of the corresponding intervention, as such it indicates an etiological pathway. Finally, Mendelian randomization studies of birth weight are open to confounding by maternal genetics causing both infant genetics and the outcomes of interest. However, the contribution of maternal genetics compared to other maternal attributes, such as nutrition, is uncertain.[13, 18]**

**To:**

**Eighth, MR provides an estimate of the effect of lifetime exposure rather than indicating the exact size of the corresponding intervention, as such it indicates an etiological pathway. Birth weight is affected by maternal and fetal genetics.[19] We used maternal genetic predictors net of infant genetics so the associations found with offspring body composition indicate the role of the intrauterine environment. Whether the intrauterine environment is a modifiable target of intervention, or whether subsequent consequences of the intrauterine environment would be more suitable for intervention requires investigation.** **Lastly, different genetic effects by generation is a concern. Given summary data was used, the genetic effects of maternal genetics net of infant genetics with offspring body composition were approximated by the genetic effects of maternal genetics net of infant genetics with maternal body composition. However, effects of genetic are likely consistent across generations.[20] We cannot rule out the possibility of the gene-environmental and/or gene-gene interactions leading to heritable epigenetic changes, which requires further exploration with individual maternal and infant genetic data.[20] ~~Finally, Mendelian randomization studies of birth weight are open to confounding by maternal genetics causing both infant genetics and the outcomes of interest.~~ ~~However, the contribution of maternal genetics compared to other maternal attributes, such as nutrition, is uncertain.[13, 18]~~**

***(Discussion-3^rd^ paragraph)***

**From:**

**Lower birth weight might indicate lower levels of androgens thus generating positive associations of birth weight with muscle mass and the stronger associations in men seen in both the observational and MR designs.**

**To:**

**Lower birth weight might indicate lower levels of androgens thus generating positive associations of birth weight with muscle mass and the stronger associations in men seen in both the observational and MR designs, although a difference by sex was not evident in the MR design.**

*(Conclusion)*

**From:**

**Higher birth weight might increase fat-free mass, potentially with stronger effects on fat-free mass in men than women.**

**To:**

**Higher birth weight might increase fat-free mass and fat mass~~, potentially with stronger effects on fat-free mass in men than women.~~**

## **C2.3**

It is also not clear to me why the authors reduced the original list of 60 BW SNPs to 47. The list of 60 loci already represents a list of independent association signals. A lot of the SNPs which were removed seem to be correlation artefacts rather than genuine LD, for example the SNP rs61830764 (DTL) is over 50mb from the nearest signal suggesting that the correlation here doesn’t reflect real LD. The signal rs1011939 (GPR139) also is the only SNP on chromosome 16 but seems to have been dropped as “not independent” although there can be no real LD with the other signals.

*Thank you for your comment.*

*Previously, we restricted our instruments to those based on people of European ancestry, which gave 47 rather than 60 SNPs. In the revised analysis we used genetic predictors from a most recent GWAS from the EGG consortium, which has decomposed the contributions of direct fetal and indirect maternal genetic effects on birth weight.[9] In total, 30 SNPs were obtained restricted to the European only meta-analysis. Restricting the two samples into the same ancestry could mitigate potential confounding bias caused by the hidden population structure.*[21]

***(Discussion-2^nd^ paragraph)***

**From:**

**Fifth, the MR study mainly pertains to people of European ancestry.**

**To:**

**Fifth, the MR study mainly pertains to people of European ancestry. However, restricting the MR study to people of European ancestry could mitigate any confounding bias caused by hidden population structure, if genetic associations vary by ethnic group.[21] Ethnic differences between the MR study and the observational study is another concern, although we usually expect causal factors to act consistently across populations, unless we have evidence that the causal mechanism differs or is less relevant in some specific populations.**

## **C2.4**

Other comments

In the limitations section, point 6 leaves me confused. They state “no age-specific genetic predictors of BW are available”, but birth weight is by definition the weight at birth, there is no age variation in BW. Point 8 also leaves me a little confused. It talks about lifetime exposure, but birth weight is again defined only at birth. I suggest the authors rephrase this point as I’m not entirely sure from what they have written what they are trying to say here.

*Thank you for your comment.*

*The point 6 is a typo, which should be updated as “sex-specific” not age-specific.*

*Although birth weight is only defined at birth, it is a proxy of intrauterine environment in this study, which is more likely to be modifiable than birth weight.*

**Reference**

1.	Mathews F, Yudkin P, Neil A. Influence of maternal nutrition on outcome of pregnancy: prospective cohort study. BMJ (Clinical research ed). 1999;319(7206):339-43. Epub 1999/08/06. PubMed PMID: 10435950; PubMed Central PMCID: PMCPMC28185.

2.	Fisher D, Baird J, Payne L, Lucas P, Kleijnen J, Roberts H, et al. Are infant size and growth related to burden of disease in adulthood? A systematic review of literature. International journal of epidemiology. 2006;35(5):1196-210. Epub 2006/07/18. doi: 10.1093/ije/dyl130. PubMed PMID: 16845132.

3.	Wang T, Huang T, Li Y, Zheng Y, Manson JE, Hu FB, et al. Low birthweight and risk of type 2 diabetes: a Mendelian randomisation study. Diabetologia. 2016;59(9):1920-7. Epub 2016/06/24. doi: 10.1007/s00125-016-4019-z. PubMed PMID: 27333884; PubMed Central PMCID: PMCPMC4970938.

4.	Yeung CHC, Au Yeung SL, Fong SSM, Schooling CM. Lean mass, grip strength and risk of type 2 diabetes: a bi-directional Mendelian randomisation study. Diabetologia. 2019;62(5):789-99. Epub 2019/02/25. doi: 10.1007/s00125-019-4826-0. PubMed PMID: 30798333.

5.	Yeung CHC, Au Yeung SL, Fong SSM, Schooling CM. Lean mass, grip strength and risk of type 2 diabetes: a bi-directional Mendelian randomisation study. Diabetologia. 2019. Epub 2019/02/25. doi: 10.1007/s00125-019-4826-0. PubMed PMID: 30798333.

6.	Zanetti D, Tikkanen E, Gustafsson S, Priest JR, Burgess S, Ingelsson E. Birthweight, Type 2 Diabetes Mellitus, and Cardiovascular Disease: Addressing the Barker Hypothesis With Mendelian Randomization. Circulation Genomic and precision medicine. 2018;11(6):e002054. Epub 2018/06/08. doi: 10.1161/circgen.117.002054. PubMed PMID: 29875125.

7.	Lopez P, Subramanian SV, Schooling CM. Effect measure modification conceptualized using selection diagrams as medication by mechanisms of varying population-level relevance. J Clin Epidemiol. 2019. Epub 2019/05/24. doi: 10.1016/j.jclinepi.2019.05.005. PubMed PMID: 31121303.

8.	Pan WH, Flegal KM, Chang HY, Yeh WT, Yeh CJ, Lee WC. Body mass index and obesity-related metabolic disorders in Taiwanese and US whites and blacks: implications for definitions of overweight and obesity for Asians. The American journal of clinical nutrition. 2004;79(1):31-9. Epub 2003/12/20. doi: 10.1093/ajcn/79.1.31. PubMed PMID: 14684394.

9.	Warrington NM, Beaumont RN, Horikoshi M, Day FR, Helgeland Ø, Laurin C, et al. Maternal and fetal genetic effects on birth weight and their relevance to cardio-metabolic risk factors. Nature genetics. 2019;51(5):804-14. doi: 10.1038/s41588-019-0403-1.

10.	Horikoshi M, Beaumont RN, Day FR, Warrington NM, Kooijman MN, Fernandez-Tajes J, et al. Genome-wide associations for birth weight and correlations with adult disease. Nature. 2016;538(7624):248-52. Epub 2016/09/30. doi: 10.1038/nature19806. PubMed PMID: 27680694; PubMed Central PMCID: PMCPMC5164934.

11.	!!! INVALID CITATION !!! [11].

12.	Howrigan D. DETAILS AND CONSIDERATIONS OF THE UK BIOBANK GWAS: THE NEALE LAB; September 20, 2017. Available from: <http://www.nealelab.is/blog/2017/9/11/details-and-considerations-of-the-uk-biobank-gwas>.

13.	Beaumont RN, Warrington NM, Cavadino A, Tyrrell J, Nodzenski M, Horikoshi M, et al. Genome-wide association study of offspring birth weight in 86 577 women identifies five novel loci and highlights maternal genetic effects that are independent of fetal genetics. Human Molecular Genetics. 2018;27(4):742-56. doi: 10.1093/hmg/ddx429. PubMed PMID: PMC5886200.

14.	Chomtho S, Wells JC, Williams JE, Lucas A, Fewtrell MS. Associations between birth weight and later body composition: evidence from the 4-component model. The American journal of clinical nutrition. 2008;88(4):1040-8. Epub 2008/10/10. doi: 10.1093/ajcn/88.4.1040. PubMed PMID: 18842792.

15.	Dodds R, Denison HJ, Ntani G, Cooper R, Cooper C, Sayer AA, et al. Birth weight and muscle strength: A systematic review and meta-analysis. The journal of nutrition, health & aging. 2012;16(7):609-15. doi: 10.1007/s12603-012-0053-9.

16.	Simpson J, Smith AD, Fraser A, Sattar N, Lindsay RS, Ring SM, et al. Programming of Adiposity in Childhood and Adolescence: Associations With Birth Weight and Cord Blood Adipokines. The Journal of clinical endocrinology and metabolism. 2017;102(2):499-506. Epub 2016/11/15. doi: 10.1210/jc.2016-2342. PubMed PMID: 27841944; PubMed Central PMCID: PMCPMC5413167.

17.	Burgess S, Davies NM, Thompson SG. Bias due to participant overlap in two‐sample Mendelian randomization. Genetic Epidemiology. 2016;40(7):597-608. doi: 10.1002/gepi.21998. PubMed PMID: PMC5082560.

18.	Du M, Yan X, Tong JF, Zhao J, Zhu MJ. Maternal obesity, inflammation, and fetal skeletal muscle development. Biology of reproduction. 2010;82(1):4-12. Epub 2009/06/12. doi: 10.1095/biolreprod.109.077099. PubMed PMID: 19516021; PubMed Central PMCID: PMCPMC2802110.

19.	!!! INVALID CITATION !!! [11-13].

20.	Nelson VR, Nadeau JH. Transgenerational genetic effects. Epigenomics. 2010;2(6):797-806. doi: 10.2217/epi.10.57. PubMed PMID: 22122083.

21.	Davies NM, Holmes MV, Davey Smith G. Reading Mendelian randomisation studies: a guide, glossary, and checklist for clinicians. BMJ (Clinical research ed). 2018;362:k601. doi: 10.1136/bmj.k601.
